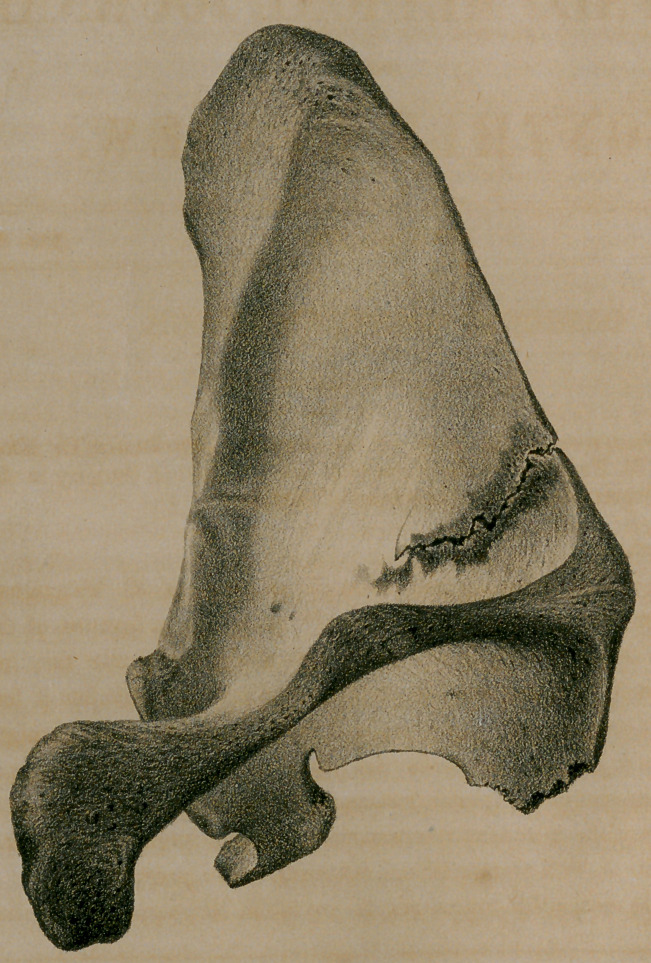# Fractures of the Body of the Scapula

**Published:** 1856-01

**Authors:** Frank H. Hamilton

**Affiliations:** Professor of the Principles and Practice of Surgery in the Medical Department of the University of Buffalo


### BUFFALO MEDICAL JOURNAL

AND

### MONTHLY REVIEW.

**VOL. 11.** JANUARY, 1856. **NO. 8.**

### ORIGINAL COMMUNICATIONS.

ART. I.—*Fractures of the Body of the Scapula.* By FRANK H. HAMILTON, M. D., Professor of the Principles and Practice of Surgery in the Medical Department of the University of Buffalo.

DOCTOR HUNT:

*Dear Sir,*—The following translation of so much of M. Malgaigne's great work on "Fractures and Dislocations,"* as relates to fractures of the *Body of the Scapula,* was made with a view solely to my own use; but when I reflected how rare was this accident, and how little attention it had received from surgical writers, I thought it worth the space which it might occupy in your journal. To make the paper more complete, however, I have added an example of partial fract:re of the body of the scapula, and a brief synopsis of the treatment recommended by other surgeons, so far as I was able to do so, with also occasional references to the prognosis.

I cannot let escape this opportunity to say of M. Malgaigne, that he has

* TRAITÉ DES FRACTURES ET DES LUXATIONS, *par* J. F. MALGAIGNE, *Chirurgien de l'Hôpital Saint-Louis, Chevalier de la Légeon-d'Honneur et du Mérite Militaire de Poligne, Membre de l' Académic Royale de Médicine. Tome* 1, *des Fractures. Avec un Atlas de* 16 *Planches, dessiné d'aprés Nature par M. Delahaye. A Paris, Chez J. B. Baillière.*

treated the whole subject of fractures with a completeness, intelligence and *honesty* which has commanded my admiration, and which I have seen exhibited by no other writer upon this department of surgery; unless, indeed, I except M. Nelaton, of whom, however, I would not speak so positively, since I have not examined his treatise with sufficient care to warrant an exact comparison.

### CHAPTER X.

### *Fractures of the Scapula.*

Fracture of the scapula is so rare that Ravaton, after a practice of fifty years, declared that he had never seen it except as produced by fire-arms upon the field of battle. Among 2358 surgical accidents reported from Hotel Dieu, only four examples are recorded. It is proper to add, however, that among 1901 fractures treated at the Middlesex Hospital, Lonsdale has noticed eighteen of the scapula.

We may distinguish four principal varieties, according as they affect the body of the bone, the acromion process, the coracoid and the glenoid cavity; but these latter being accompanied generally with a displacement of the head of the humerus, their history will be more properly referred to the history of dislocations.

### § I. *Fractures of the Body of the Scapula.*

It has been sought to establish among these fractures several varieties. At first J. L. Petit divided them into transverse, oblique and longitudinal. Later, Desault mentioned as a distinct species, fracture of the inferior angle; Bottcher imagined fractures of the posterior angle; A. L. Ritcher has reproduced, after Paul of Egineta and Ambrose Pare, fracture of the spine of the scapula. I know of no example of a fracture limited to the spine, or to the posterior angle, nor indeed of a vertical fracture; fracture of the inferior angle does not merit special mention; and in short the actual varieties and those which it is important to distinguish, are incomplete fractures, complete transverse or oblique, and multiple or comminuted.

They are generally produced by a direct cause, a blow, the fall of a heavy body upon the scapula, or the fall of the person himself backward. Doctor Heylen has, however, recently published a case of fracture attributed to muscular action. A man, forty-nine years old, wishing to mount into his cart, seized upon its side with his left hand, at which moment, while suspended by this hand, the horse started into a brisk trot. The man was thus carrie d hanging by his left arm, a distance of one hundred meters, until the horse stopped. In addition to an acute pain in his left shoulder, aggravated by the least movement, the finger detected a depression in the middle of the spine of the scapula; and on pressing forcibly upon the projecting fragment it receded from the finger with a sensation of crepitus. Moreover, there was not the slightest degree of ecchymosis perceptible externally, and the patient, who had preserved completely his presence of mind, declared that nothing had touched his shoulder. Perhaps the accident ought to be charged to the weight of the body, augmented by the jolting of the carriage, and which may have broken the bone by a sort of extension; but whatever interpretation is given, the fact is not the less remarkable.

I have never seen but one incomplete fracture, and I know of no other example.* A terracemaker, at work in an excavation, and while bending forward, received upon his back and left shoulder blade, a rough block of

---

* I have been unable to find any other example of incomplete fracture of the scapula than this one mentioned by M. Malgaigne. I have, therefore, had executed upon stone, a drawing of a specimen contained tn my own cabinet. The bone was presented to me by Dr. N. C. Powers, of Syracuse, and belongs to the skeleton of the Oneida Indian "Nimham," who was a great fighter, and who died when about 45 years old, in consequence of severe injuries received in a street brawl; but his death did not occur until four or five months after the receipt of the injuries.

In addition to this fracture of the right scapula, five of his ribs were broken and both legs, all of which, except the scapula, had united completely by intermediate and ensheathing callus. He was under the care of Dr. Morrison, of Oneida Castle.

The lithograph has been drawn with great fidelity, by Charles E. Lewis, lithographer, of this city.

The scapula (see engraving) is broken nearly transversely; commencing upon the posterior margin at a point about three-quarters of an inch below the spine, and extending across the body of the bone one inch and three-quarters, in a direction inclining a little upward, irregularly denticulate and without comminution. The fragments are in exact apposition, and throughout most of their extent in immediate contact. They are, however, not consolidated at any point, but upon either side of the fissure there is a ridge of ensheathing callus, of from one to three or four lines in breadth, and of half a line or less in thickness along the broken margin, from which point it subsides gradually to the level of the sound bone. The same is observed upon the inner as well as upon the outer surface of the scapula. This callus has assumed the character of complete bone, but it is more light and spongy than the natural tissue, and the outer surface has not yet become lamellated. Its blood canals and bone cells open everywhere upon the surface.

Directly over the fracture, and between its opposing edges, no callus exists, but as the bone had lain some time in the earth before it was exhumed, it is probable that a less completely organized intermediate callus had occupied this space, and stone, weighing 10 kilogrammes, which had fallen from a height of 4 or 5 metres. Upon examination I found a severe contusion near the center of the fossa infra spinatus; the finger settling into a very marked depression bounded within by a firm bony projection, and which ascended gradually outward to the level of the rest of the bone. The scapula moved entire and without crepitus. This was, then, a fracture with depression of the infra spinatus fossa. It will be understood that in a case like this the role of art is very limited; I contented myself with holding the arm snugly against the trunk, that I might insure immobility of the scapula until the pain had entirely ceased.

Complete fractures, transverse or oblique, occur most often below the spine. They are sometimes without displacement. M. Huguier mentioned to me that he had treated one of this kind, which he recognized by the crepitus, conjoined with a slight mobility. Kirkbride has published an account of a man who, thrown backward by the blow of a machine upon the iron track of a railroad, received a transverse fracture at a point 6 or 8 centimetres below the spine; the fragments were easily displaced, but they returned in contact as soon as they were left to themselves. The man having died on the fifty-fourth day, the fracture was found firmly reünited, with a callus extending across the bone.*

But ordinarily there exists a displacement more or less considerable, as the combined result of the external violence and of muscular action. Fig. 3, pl. 4, represents the left scapula of a young epileptic, who a long time before his death had this bone fractured, nearly transversely at two points; in the first, situated below the spine, the inferior fragment has been subjected to a triple displacement, first forward, then upward by an actual overriding, and, finally, outward. The second, situated near the inferior angle, presents the same displacements, only a little more marked; and the overriding of the two fragments is such that the scapula has lost in height fifteen millimetres.

I have seen a displacement a little different in an old man of seventy-two years, who had been thrown down by a cabriolet. We detected a fracture

---

that, owing to the less proportion of earthy matter which it contained, it had become decomposed and had been removed.

One may notice here: *First,* the existence of true ensheathing callus in a flat bone; *second,* its presence where there could have been no motion of the opposing fragments, and consequently no *necessity* for a "provisional splint." F. H. H.

* American Journal of Medical Sciences. August, 1855, p. 307.

of the right scapula, which was treated by a single bandage around the body. On the thirty-fourth day, the consolidation appearing to be complete, I examined the condition of things carefully. The fracture divided transversely the external half of the fossa infra spinatus, then ascended a little obliquely inward toward the spinal margin. The inferior fragment was carried very perceptibly outward; but instead of having been thrust forward, it projected backward; and the finger sliding from above downward upon the side of the scapula, was arrested by a jutting of this fragment quite marked, elevating the infra spinatus muscle. Nevertheless the two fragments were not comcompletely separated, for a measurement of the two scapulæ did not show the least indication of an overlapping.

Lonsdale reports two cases of simple oblique fractures, but with very slight displacement; the inferior fragment being carried forward and outward, and the other overriding it. When the fragments are separated by more considerable external violence the displacements take a new character, which it will be found more difficult to explain. Figures 1 and 2 of plate four, represent the two faces of a scapula broken into numerous fragments; and one of the fractures divides the infra spinatus fossa obliquely, from below upward and from within outward. But instead of descending and riding upon the other, the superior fragment is carried backward; the inferior is drawn outward, in such a manner as that while they remain in contact near the neck of the scapula, they separate downward and backward like the two branches of a compass. The patient had survived the accident some time; and consolidation, commenced at different points, had fixed the principal fragments in the relations seen in the two engravings. It will be seen, also, that the inferior fragment, which includes the entire angle, is carried before and without the middle fragment upon which it rides, the same as in figure 3 already described. Probably there had existed very extensive muscular lesions; and perhaps we ought not to expect ever to meet with similar phenomena except in comminuted fractures.

The symptoms are generally: a local pain augmented by pressure, sometimes by coughing and sneezing, or especially by the motions of the arm; communicated movements producing much less pain than voluntary movements. With one of my patients the pain was so severe as to render voluntary movements almost impossible. Sometimes the head inclines to that side, as in fractures of the clavicle. In one instance I observed a considerable ecchymosis, which I have not met in any other case. J. L. Petit adds that emphysema is almost always present. I do not know that any one except himself has repeated this observation. So far the symptoms might only lead one to suspect a fracture; the only reliable diagnostic signs are crepitation, mobility, and, finally, displacement.

Crepitation is not easily obtained by direct friction of the two fragments, which offer to the hands so little hold; but it is produced by inflicting upon the arm and the shoulder extensive motions upward, downward, forward, backward, at the same time that the hand is applied flat upon the scapula to detect the crepitation.

Mobility is not much present except where the fracture is accompanied with displacement. In fractures of the inferior angle, Desault carried the shoulder, and, consequently, the scapula, backward, while he supported his fingers upon the suspected angle. If the angle does not follow the movements of the rest of the bone, it is conclusive evidence of a fracture; but when it does follow, one ought not to say, with Bichat, that there is no fracture; the only legitimate inference must be that there is no displacement.

Even the displacement is not always easy to appreciate, especially in fat and muscular subjects, and even though there may be ever so little tumefaction. We may bring sufficiently into view the projection of the spinal border by making the patient cross the arms upon the chest. Another method which I employ with advantage, is the following: the forearm is carried behind the back and the hand is then lifted as high as possible; in this position the scapula detaches itself like a wing from the surface of the thorax, and its spinal border, its inferior angle and its external border, lift the integuments and show almost in relief all their abnormal projections. In this manner, also, we can the better seize upon the different parts of the bone and attempt to make them play one upon the other to obtain crepitation. It is, nevertheless, well to be on our guard when we examine the fossa infra spinatus, not to be deceived by the natural projection outward of the side of the scapula; by the prominence above of the spine and its root, or by the relief of the spinal border near the inferior angle. In case of doubt we ought to examine the two scapulas in the same position, and compare carefully all their projections.

With all these precautions we can easily determine the existence of a fracture; but to know whether it is transverse or oblique, simple or multiple, is another thing. Often, indeed, when the displacement is nothing, or very inconsiderable, crepitation will declare a fracture to which the touch cannot assign its place; and, finally, a fracture without displacement, and without crepitation, will be almost inevitably unrecognized.

It is fortunate, then, that in these cases the mistake is without importance; and the prognosis is scarcely more grave in fractures which are accompanied with the most striking displacement. B. Bell affirms that they determine, very frequently, in the movements of the arm a permanent stiffness;* but for myself, I have seen nothing of this kind, and I have not even remarked an appreciable constraint in the movements with the subjects whom I have seen.

Fractures without displacement require nothing but repose, and it is sufficient to hold the arm snug against the trunk with a body-bandage and a sling.

When there exists displacement, the reduction has been effected in various ways. Pierre d'Argelata places a ball under the arm and brings again the elbow against the ribs. J. L. Petit directed that the arm of the patient should be lifted until the fold of the elbow was opposite the nose; and, while an assistant maintained it in this position, the surgeon should adjust the fragments as well as possible. B. Bell recommended to elevate the head and the shoulders to relax the muscles of the back. Heister drew the arm forward;† Desault, for fractures of the inferior angle, carried the arm before the chest, holding it a little removed from the body, while the hand of the injured side was placed upon the extremity of the sound shoulder.‡

Surgeons are not much better agreed as to the apparel to be employed. Paulus, of Argineta, treated these fractures as those of the clavicle, recommending that the patient should be kept reclining upon the sound side. Albucasis applied upon the scapula a sort of *etoupade*, above which again are placed compresses, and splints of wood or of leather. Desault employed a wedgeshaped cushion, of which the apex corresponded to the axilla, and the base to the chest, in order to furnish a *point d'appui* for the arm; the whole supported by a bandage six or seven ells in length, of which the first turn was intended to fix the hand of the injured side upon the sound shoulder. Boyer,‖ without having any regard to the displacement, occupied him-

---

* "It is always difficult to cure, and induces commonly a permanently stiff and unwieldy state of the corresponding arm."—*System of Surgery, by Benjamin Bell, p.* 478, 3*d Philad'a Ed.* F. H. H.

† Heister adds, moreover, that we ought to apply proper compresses and slips of pasteboard wet "*cum spiritu vini vel oxyerato*," and firmly bound on with the stellate or four-headed bandage.—*Institutiones Chirurgical de D. Laurentii Heisteri. Pars Prima p.* 94, *Ed. Amstelaedami*, 1739. F. H. H.

‡ Treatise on Fractures, Luxations, &c., by P. J. Desault, p. 64. Philad'a Ed. 1805. F. H. H.

‖ For vertical fractures, or transverse and through any part considerably above self in maintaining the immobility of the bone; and, therefore, by appropriate turns of the bandage he secured the arm closely to the side of the body, carrying the elbow at the same time a little forward.

Among such a diversity of practice, which shall the surgeon choose? shall he attempt the reduction, and have we the means to accomplish it?

The three indications to fulfill will be to carry the inferior fragment backward and inward, the superior forward and outward, and lastly, to correct the overriding. The inferior fragment seems to be drawn away by the teres major; it is necessary, then, in order to relax this muscle, to approach the arm to the trunk, and even to incline it a little backward. The superior fragment appears to be under the predominating influence of the rhomboid, which is relaxed by carrying the shoulder upward and backward. But as to the overriding, I do not see any means by which it can be overcome.

Position alone will not suffice to correct the two first displacements; it will be found necessary to employ also adjustment with the hands, and farther, the retentive apparatus must combine at the same time: *first*, means capable of holding the shoulder upward and backward with the elbow near the body; and upon this point the reader may consult what we have said upon the subject of this indication when speaking of fractures of the clavicle: *second*, of means suitable to replace the pressure of the hands — a compress upon the superior fragment to press it forward against the other — graduated compresses beneath the same to hold it outward, and upon the outer side of the inferior fragment to hold it inward.

Such, at least, are the means which a study of the actual displacements, and their most probable explanations, would seem to indicate; but here, as in many other cases, nature makes light of our theories; and for myself I confess that in the few fractures of this kind that I have had occasion to treat, I have not been able, by any of the means which I have indicated, to reduce the displacements, much less to maintain the reduction. Indeed I must say that the positions which seem the most rational, sometimes increased the displacement, which was again diminished by other attitudes, variable for each subject. If, then, a surgeon shall ask advice with regard

---

the lower angle, Desault's apparatus without the axillary cushion. To place the hand upon the opposite shoulder, is, however, "unnatural and fatiguing." In fracture of the inferior angle, "the arm is to be pushed inward, downward and forward, the forearm being half bent." The arm is to be retained in this position by a body bandage, seven yards long; the lower fragment is to be supported by a compress and an additional roller, with a sling.—*Boyer on the Bones, p.* 70, *Philada Ed.* 1805.

F. H. H.

to the reduction, the only counsel which experience authorizes me to give to him, will be to try every possible attitude until he has found the best, and then to maintain this position as long as may be necessary for consolidation.

But in the major part of these fractures, such excessive care is not demanded; and in others, the reduction, often so impossible to accomplish, is of no very great importance, and I content myself with holding the arm up with an ordinary sling while it is kept snugly against the trunk with a body-bandage.

*Prognosis established by other surgeons; with also a brief synopsis of their plans of treatment. Compiled by the Translator.*

*Boulton.* If the scapula "be broke in several pieces which are not likely to unite, Incision is to be made, and the loose pieces are to be taken out, leaving the rest to nature." "If the scapula be broke near the Joint, it is for the most part incurable."—*System of Chirurgery, by Richard Boulton, p.* 324. *London:* 1713.

*Turner.* Where the patient is not so fat or so muscular as to render it impracticable, the fingers must be thrust underneath, so as to elevate the depressed fragments, "which having replac'd, with suitable Bolstering and Deligation, you are, as much as possible, to keep it up." A careful regimen is to be enjoined, with rest and proper therapeutics, "and when he has done this, as becomes him, I see not any Injustice, that he should be paid for a Cure, in which, if the Work succeed, *Nature* rather than *Art* had the chiefest Hand."—*Art of Surgery, by Daniel Turner, vol.* 2, *p.* 260. *London Ed.* 1742.

*B. Bell.* Elevate the head and shoulders, to relax the muscles of the back, and at the same time support the humerus so as to relax the deltoid. Retain the arm and shoulder in this position by suitable rollers.—*System of Surgery, by Benjamin Bell, p.* 478. *3d Philad'a Ed.* 1806.

*Amesbury.* When the body is broken, "it is merely necessary to fix the arm to the side by means of a bandage, which includes the arm from the shoulder to the elbow." When the inferior angle is broken off the arm is to be pushed "inward, downward and forward, where it is to be kept by a roller. The fragment is also to be kept backward as much as possible, with compresses and a roller. The arm is to be supported in a sling."—*On Fractures, by Joseph Amesbury, vol.* 2, *p.* 534. *London Ed.* 1831.

*Liston.* "It is sufficient to restrain motion; and this is effected by passing a bandage round the chest, over the scapula, and round the arm."—*Elements of Surgery, by Robert Liston, p.* 470. *Philada Ed.* 1837.

*Lonsdale.* The elbow is to be lifted by a sling, and the arm and body are to be swathed with a broad roller. If the lower portion is disposed to become displaced, it may be supported by a pad. "It is seldom, however, that much advantage can be gained by this." * * * Little harm results if the fractured portions unite in the position into which they are driven at the time of the accident. * * * The free use of the shoulder joint, however, is not recovered till some time after."—*Treatise on Fractures, by Edward F. Lonsdale, p.* 191. *London Ed.* 1838.

*S. Cooper.* "When the lower angle of the scapula is broken off, the humerus may be brought forward across the chest and the hand confined upon the opposite shoulder." "This position is adopted abroad; but, in this country, when any part of the body of the scapula is fractured, we merely apply the spica bandage." Mr. Cooper regards this bandage as "of little or no use," and speaks of the sling which is employed with it, as "the efficient part of the apparatus."—*First Lines of Surgery, by Samuel Cooper, vol.* 2, *p.* 327. *New York Ed.* 1844.

*South.* A simple roller to keep the arm against the body, and a sling.—*Note to Chelius, p.* 602.

*Skey.* A sling, with a bandage to confine the arm to the body, or with Desault's apparatus.—*Operative Surgery, by Frederick C. Skey, p.* 157. *Philad'a Ed.* 1851.

*B. Cooper.* When the dorsum is broken Mr. B. Cooper employs a long roller which is made to cover the arm and secure it to the chest; but when the fracture is at the inferior angle, he recommends that the arm be carried across the chest.—*Surgery, by Bransby B. Cooper, p.* 227. *Philad'a Ed.* 1852.

*Erichsen.* "A body-bandage."—*Surgery, by John Erichsen, p.* 206 *Amer. Ed.* 1854.

*Lizars.* "A broad flannel bandage, or riding belt, should be put around the body, and the arm bound to the side." ("It is with difficulty retained *in situ.*")—*Practical Surgery, by John Lizars, p.* 132. 2*d Edinburgh Ed.* 1847.

*Miller.* "It is sufficient to restrain motion, by wearing the arm in a sling, and by having a broad flannel bandage passed tightly over the chest, including the fractured bone."—*Practice of Surgery, by James Miller, p.* 311. *Philad'a Ed.* 1853.

*Pirrie.* When the angle is broken off, or even though the fracture be considerably above the angle, if it is complete and transverse, it will be necessary to place one compress in front of the lower fragment, and one behind the upper, and then having pressed the arm downward, forward and inward, to secure it with a roller, &c. If the body is broken otherwise, it will be sufficient to put the arm in a sling and to secure the arm to the body with a roller.—*System of Surgery, by William Pirrie, p.* 146. *Philad'a Ed.* 1852.

*Gorter* recommends in all fractures, whether of the body, processes or neck, that in order to accomplish the reduction, the elbow shall be carried from the body to relax the deltoid, and that the arm be then suspended and kept at rest in a sling. ("*In restitutione attollendum brachium, ut Deltoides musculus sit laxus, dum digitis Chirurgi reponatur fractura. Post repositionem diu non debet aeger conari elevare brachium, sed gerere debet brachium mitella admodum sustentatum.*")—*Chirurgia Repurgata, auc Johannis de Gorter. Lugduni Batavorum, p.* 80. 1742.

*Chelius.* Confinement of the arm to the side of the body, with a conical pad in the axilla the apex of which should correspond to the axilla; the elbow lifted by a sling, or bandage, or both; a figure of 8 bandage upon the shoulder. Where the fracture is transverse and considerably above the angle, the hand may rest, if the patient can endure it, upon the sound shoulder. ("If the fracture is near the angle the cure is always effected with some deformity: but which does not interfere with the motions of the arm.")—*Chelius' Surgery, vol.* 1, *p.* 601. *American Ed.* 1847.

*Tavernier.* In fractures of the body he employs a simple bandage to confine the arm to the side of the body. In fractures of the angle the upper fragment is to be carried backward and downward by bringing the elbow forward and upward; and the whole is to be secured by a long roller.—*Operative Surgery, by A. Tavernier, p.* 378. *Philad'a Ed.* 1829.

*Nelaton.* Rejecting all the complicated forms of apparatus, which he believes to have been proven inefficient, M. Nelaton remarks, "In truth we are forced to acknowledge that we have not any means to act upon the fragments; all that we can do is to maintain their immobility, and upon this occasion *I will repeat what I said when treating of fractures of the clavicle; all our retentive means being equally insufficient, the best will be that which will cause the least pain;* a simple sling, which will embrace the elbow, the arm and the forearm, will then suffice. I must, however, give the preference to the bandage of M. Mayor, because it maintains the shoulder more firm." —*Elémens de Pathologie Chirurgicale, par A. Nelaton. Tom. premier, p.* 723. *Paris Ed.* 1844.

*Mayor* employs the same apparatus which is recommended by him for fractured or dislocated clavicle, namely, a sling with two broad shoulder straps, which are made to rest respectively upon the two shoulders. The elbow being carried forward across the body.—*Nouveau System de Delegation Chirurgicale, par Matthias Mayor de Lausanne. Troisieme Ed., p.* 396. *Paris:* 1838. *See also Figs.* 23 *and* 24, *Plate* 3.

*Gibson.* In case of fracture of the lower angle, a thick compress is to be placed in front of the lower fragment and then retained by an additional roller or a sling. * * * "It is hardly possible to restore the fragment to its former position." * * * Yet, they "may be made to approximate so closely as to leave little or no deformity." "Sometimes the patient recovers sooner when confined to bed during the whole treatment."—*Surgery, by William Gibson, vol.* 1, *p.* 278. 6*th Philad'a Ed.* 1841.

*Sargent.* A broad compress and a wide roller around the chest, with the arm in a sling and confined to the chest. If the inferior angle is broken, "the arm should be carried backward" and confined by a broad roller with compresses, so applied as to support both fragments of the scapula. The sling must be used here also.—*Minor Surgery, by F. W. Sargent, p.* 153. *Philad'a Ed.* 1848.

## Figures and Tables

**Figure f1:**